# Leptogorgins A–C, Humulane Sesquiterpenoids from the Vietnamese Gorgonian *Leptogorgia* sp.

**DOI:** 10.3390/md18060310

**Published:** 2020-06-13

**Authors:** Irina I. Kapustina, Tatyana N. Makarieva, Alla G. Guzii, Anatoly I. Kalinovsky, Roman S. Popov, Sergey A. Dyshlovoy, Boris B. Grebnev, Gunhild von Amsberg, Valentin A. Stonik

**Affiliations:** 1 G.B. Elyakov Pacific Institute of Bioorganic Chemistry, Far Eastern Branch of the Russian Academy of Sciences, Pr. 100-let Vladivostoku 159, 690022 Vladivostok, Russia; ikapust@rambler.ru (I.I.K.); gagry@rambler.ru (A.G.G.); kaaniv@piboc.dvo.ru (A.I.K.); prs_90@mail.ru (R.S.P.); dyshlovoy@gmail.com (S.A.D.); grebnev_bor@mail.ru (B.B.G.); stonik@piboc.dvo.ru (V.A.S.); 2 Department of Oncology, Hematology and Bone Marrow Transplantation with Section Pneumology, Hubertus Wald-Tumorzentrum, University Medical Center Hamburg-Eppendorf, 20251 Hamburg, Germany; g.von-amsberg@uke.de; 3 Martini-Klinik, Prostate Cancer Center, University Hospital Hamburg-Eppendorf, 20251 Hamburg, Germany

**Keywords:** gorgonian, *Leptogorgia*, humulane sesquiterpenoids, anticancer activity

## Abstract

Leptogorgins A–C (**1**–**3**), new humulane sesquiterpenoids, and leptogorgoid A (**4**), a new dihydroxyketosteroid, were isolated from the gorgonian *Leptogorgia* sp. collected from the South China Sea. The structures were established using MS and NMR data. The absolute configuration of **1** was confirmed by a modification of Mosher’s method. Configurations of double bonds followed from NMR data, including NOE correlations. This is the first report of humulane-type sesquiterpenoids from marine invertebrates. Sesquiterpenoids leptogorgins A (**1**) and B (**2**) exhibited a moderate cytotoxicity and some selectivity against human drug-resistant prostate cancer cells 22Rv1.

## 1. Introduction

Marine gorgonian corals have been reported to be a rich source of isoprenoids with unprecedented chemical structures and biological activities [1]. Species of the genus *Leptogorgia* (Gorgoniidae) have been shown to produce cembranoids [2,3,4,5,6,7], polyoxygenated steroids [8,9,10,11,12], alkaloids [13], fatty acids [14], homarine [15], thyroxine, and vitamin D [16]. To date, different humulane-type sesquiterpenoids have been found in plants [17,18,19], liverworts [20], and fungi [21,22,23]. However, until recently they were not found in marine invertebrates, including gorgonians. Interestingly, two new norhumulene were isolated from the soft coral *Sinularia hirta* [24]. In addition, one more norhumulene was found in a formazan soft coral *Sinularia gibberosa* [25]. Humulanes from the peeled stems of *Syringa pinnatifida* inhibit NO production in LPS-induced RAW264.7 macrophage cells and decrease the TNF-α and IL-6 levels in RAW264.7 cells [26]. Additionally, plant cytochrome P450 was reported to catalyse the conversion of α-humulene into 8-hydroxy-α-humulene [27].

**Figure 1:**
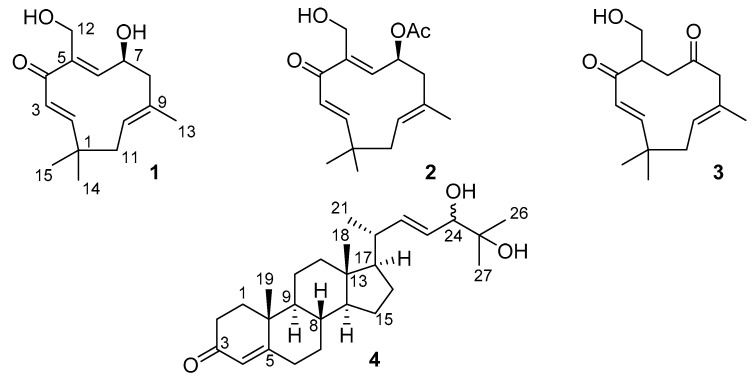
The structures of **1**−**4**.

For some humulenes, an antitumor activity was reported. Thus, zurumbone (2,6,9-humulatriene-8-one), as an active component of the *Zingiber aromaticum* extract, was shown to be active in human cancer HT-29, CaCO-2, and NCF-7 cell lines. Remarkably, it was more active than curcumin, which was used as a reference compound [28]. Herein, we report the structures and biological activities of three new humulane sesquiterpenoids, leptogorgins A–C (**1**–**3**), and a new steroid, leptogorgoid A (**4**), from the gorgonian *Leptogorgia* sp. (Figure 1).

## 2. Results and Discussion

The EtOH extract of the gorgonian *Leptogorgia* sp. (registration number O38-011) was concentrated and partitioned between aqueous EtOH and *n*-hexane. The EtOH-soluble materials were separated by silica gel flash chromatography, followed by Sephadex LH-20 column chromatography and normal and reversed-phase HPLC to give leptogorgins A–C (**1**–**3**, 2.5, 0.8, and 1.0 mg, respectively) and leptogorgoid A (**4**, 0.6 mg). 

**Figure 2:**
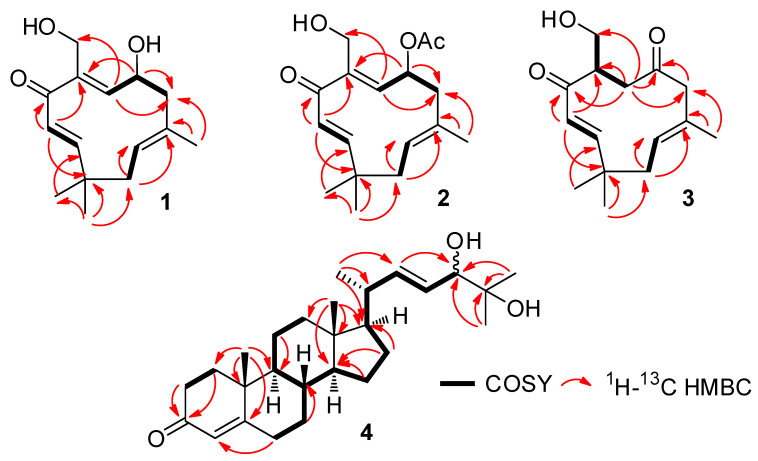
Selected COSY and HMBC correlations for **1**–**4**.

Compound **1** was isolated as a colourless oil. The HRESIMS of **1** showed an [M + Na]^+^ ion peak at *m/z* 273.1459 and an [M − H]^−^ ion peak at *m/z* 249.1498, which indicated a molecular formula of C_15_H_22_O_3_. The ^13^C NMR spectrum displayed 15 signals, which could be assigned to a sesquiterpene substructure. Analysis of the ^1^H, ^13^C, and HSQC NMR spectra (Table 1) revealed signals indicative of one ketocarbonyl (δ_C_ 200.8, C-4), one oxymethine (δ_H_ 4.21/δ_C_ 71.7, C-7), one oxymethylene (δ_H_ 4.25; 4.38/δ_C_ 64.7, C-12), four methines (δ_H_ 6.32/δ_C_ 164.8, C-2; δ_H_ 5.97/δ_C_ 128.1, C-3; δ_H_ 5.75/δ_C_ 133.8, C-6, and δ_H_ 5.22/δ_C_ 125.9, C-10), two quaternary (δ_C_ 143.0, C-5; δ_C_ 132.4, C-9) olefinic carbons, and two methylene groups (δ_H_ 1.96 and 2.68/δ_C_ 45.3, C-8; δ_H_ 1.95 and 2.40/δ_C_ 40.7, C-11), as well as one quaternary carbon (δ_C_ 38.0, C-1), one corresponding vinylic methyl (δ_H_ 1.72/δ_C_ 20.1, CH_3_-13) and two methyl singlets (δ_H_ 1.18/δ_C_ 24.0, CH_3_-14; δ_H_ 1.13/δ_C_ 29.1, CH_3_-15). The ^1^H-^1^H COSY spectrum enabled three structural fragments to be established: CH=CH-, -CH-CH-CH_2_-, and -CH-CH_2_-, which could be connected by observing the correlations in the HMBC experiment (Figure 2). Thus, HMBC correlations from H-3 to C-1, C-4, and C-5, from H-6 to C-12 and C-8, from H-7 to C-5 and C-8, from H-8 to C-7, C-9, C-10, and C-13, from H-11 to C-10, C-9, and C-1, and from CH_3_-14 and CH_3_-15 to C-1, C-2, and C-11 established the planar structure of **1** (Figure 2). 

**Figure 3:**
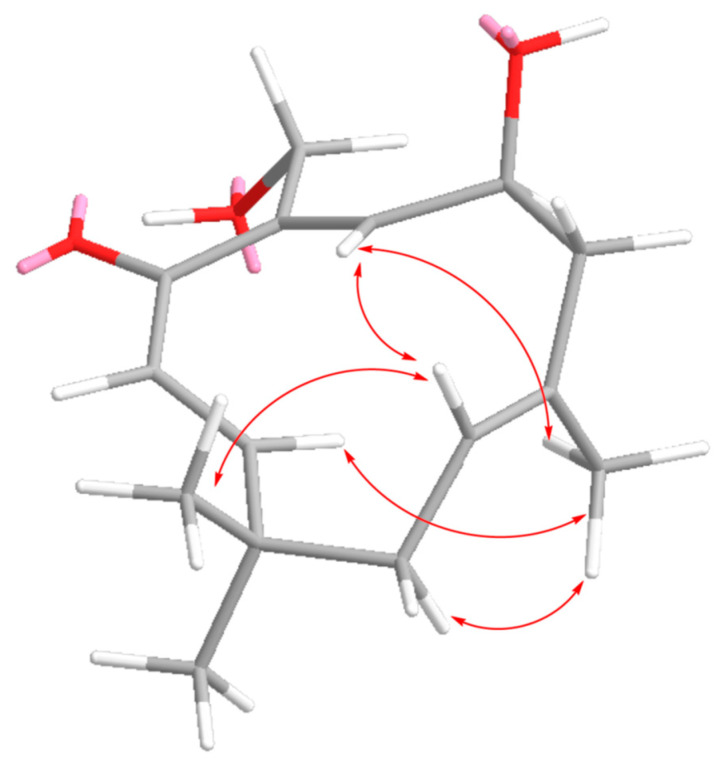
Key NOE correlations for **1**.

The geometry of the Δ^2,3^ double bond was further determined to be *E* by considering the coupling constant (*J* = 16.3 Hz) displayed in its ^1^H NMR spectrum. The NOE correlations of CH_3_-13 to H-2, H-6, and CH_2_-11, as well as H-10 with H-6 and H-6 with H-2 (Figure 3), suggested that the Δ^5(6)^ and Δ^9(10)^ double bonds in **1** were *E* configured.

**Figure 4:**
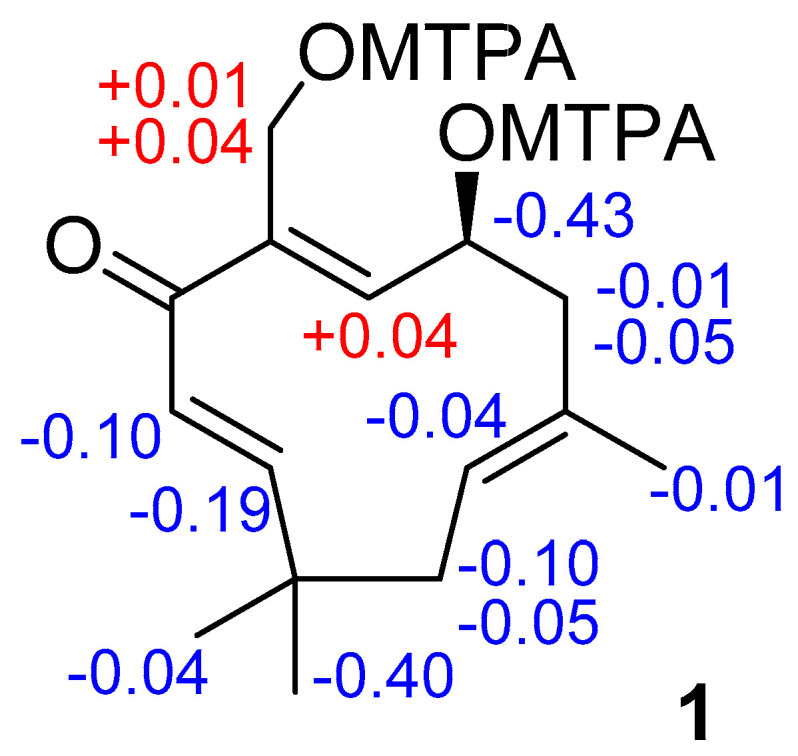
Δδ (δ^S^ − δ^R^) values (in ppm, CDCl_3_) for the MTPA esters of **1**.

A modified Mosher ester analysis was obtained, and the negative Δδ^SR^ (δ^S^ − δ^R^) values of Ha-8, (Δδ_H_ −0.01), Hb-8, (Δδ_H_ −0.05) and CH_3_-13 (Δδ_H_ −0.01), and positive Δδ^SR^ values of H-6 (Δδ_H_ +0.04) Ha-12 (Δδ_H_ +0.01), and Hb-12 (Δδ_H_ +0.04) (Figure 4) revealed the 7*S* configuration [25]. Thus, the structure of **1** was determined as 4-oxohumula-2*E*,5*E*,9*E*-trien-7*S*,12-diol, as shown in Figure 1, and named leptogorgin A (**1**). 

Compound **2** was obtained as a colourless oil. The HRESIMS of **2** showed an [M + Na]^+^ ion peak at *m/z* 315.1567 and an [M − H]^−^ ion peak at *m/z* 291.1602, which indicated a molecular formula of C_17_H_24_O_4_. The ^1^H and ^13^C NMR spectra of **2** (Table 1) were similar to those of **1**, suggesting that this compound possessed the same humulane skeleton. The key differences were in δ_H_ for H-7 and δ_C_ for carbon 7 in the spectrum of **2** (δ_H_ 5.28/δ_C_ 72.9). The corresponding signals were shifted downfield, compared to those of **1** (δ_H_ 4.21/δ_C_ 71.7). This characteristic difference and HRESIMS data were caused by the hydroxy group in **1** being displaced by an acetoxyl group in **2**. The HMBC spectra of **2** demonstrated the expected key correlations. The ECD spectrum of compound **2** was compared with the ECD spectrum of leptogorgin A (**1**), in which the corresponding absolute configuration was established by modification of Mosher’s method. Both ECD spectra displayed similar Cotton effects (see Figure S27), allowing us to establish the same 7*S* configuration for compound **2**. From these data, compound **2** was determined to be 4-oxohumula-2*E*,5*E*,9*E*-trien-7*S*-acetate,12-ol, as shown in Figure 1, and named leptogorgin B (**2**). 

Compound **3** was isolated as a colourless oil. The HRESIMS of **1** showed an [M + Na]^+^ ion peak at *m/z* 273.1459 and an [M − H]^−^ ion peak at *m/z* 249.1496, which indicated a molecular formula of C_15_H_22_O_3_. The ^1^H and ^13^C NMR spectra (Table 1) of **3** were similar to those of **1** and **2**, suggesting that this compound also possessed the same humulane skeleton. Key differences concerned δ_H_ for protons 6, 7, and 8 and δ_C_ for carbons 4, 5, 6, 7, and 8 in the spectrum of **3**, which were different compared to those of **1** and **2**. This characteristic difference was caused by an absence of the hydroxy group, as in **1**, or acetyl, as in **2** at position 7, being displaced by a ketogroup in **3**, as well as by the absence of the 5,6 double bound in **3**. The location of the ketogroup was further determined to be at C-7 by COSY, HSQC, and HMBC experiments. Thus, compound **3** was determined to be 4,7-dioxohumula-2*E*,9*E*-dien-12-ol, as shown in Figure 1, and named leptogorgin C (**3**).

Compound **4** was isolated as a colourless powder. The HRESIMS of **4** showed an [M + Na]^+^ ion peak at *m/z* 437.3026 and an [M − H]^−^ ion peak at *m/z* 413.3061, which indicated a molecular formula of C_27_H_42_O_3_. The data of 1D- and 2D-NMR spectra of **1** (Table 2) indicated that this compound belonged to steroids. Its spectra contained five methyl groups, including two angular methyl groups in the steroid nucleus (δ_H_ 0.74/δ_C_ 12.2, δ_H_ 1.19/δ_C_ 17.4) and three methyl groups of the side chain (δ_H_ 1.04/δ_C_ 20.3, δ_H_ 1.15/δ_C_ 23.8, and δ_H_ 1.20/δ_C_ 26.4), eight methylene groups, six methine groups, including one oxygenated methine (δ_H_ 3.85/δ_C_ 79.7), two quaternary sp^3^ carbons (δ_C_ 38.6, δ_C_ 42.5), one quaternary sp^3^ oxygenated carbon (δ_C_ 72.8), one trisubstituted double bond (δ_H_ 5.72/δ_C_ 123.8 and 171.4), one disubstituted double bond (δ_H_ 5.61/δ_C_ 140.8 and δ_H_ 5.43/δ_C_ 126.0), and one conjugated with double bond ketone carbonyl (δ_C_ 199.5). The geometry of the 22,23 double bond was further determined to be *E* by considering the coupling constant (*J* = 15.3 Hz) displayed in its ^1^H NMR spectrum. The HMBC spectra of **4** demonstrated the expected key correlations. From these data, compound **4** was determined to be 3-oxocholesta-4*E*,22*E*-diene-24,25 dienol, as shown in Figure 1, and named leptogorgoid A (**4**). 

**Figure 5:**
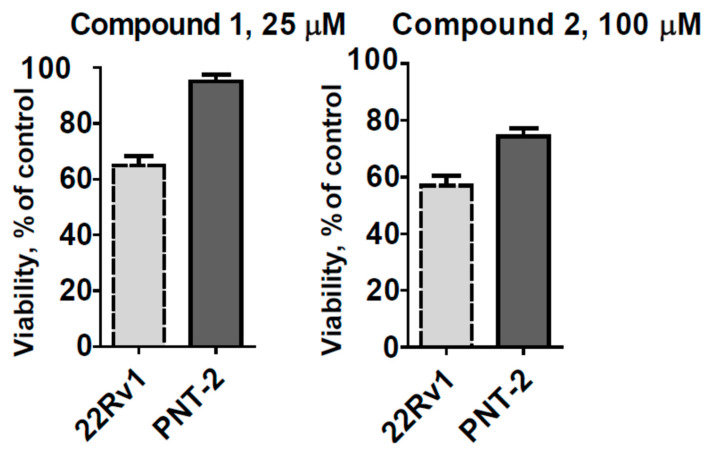
The viability of 22Rv1 and PNT2 cells after 72 h of treatment with the indicated concentrations of the investigated compounds. The viability was evaluated using MTT assay.

Next, we investigated the effects of the leptogorgins A (**1**) and B (**2**) on the viability of 22Rv1 cells (human drug-resistant prostate cancer cells) as well as on PNT2 cells (human prostate non-cancer cells). MTT assay revealed **1** to exhibit a moderate cytotoxicity to both cell lines (IC_50_ = 31.0 µM and 35.8 µM, respectively), whereas **2** had IC_50_ > 100 µM. Doxorubicine was used as a positive control and exhibited in 22Rv1 and PNT2 cells IC_50_ of 0.084 µM and 1.12 µM, respectively. Interestingly, both compounds were more active in human cancer 22Rv1 cells, in comparison with PNT2 cells (Figure 5). Additionally, we examined the ability of these compounds to inhibit the colony formation of 22Rv1 prostate cancer cells; however, no significant inhibitory activity was observed under the treatment with cytotoxic or non-cytotoxic concentrations of the compounds up to a concentration of 100 µM (data not shown). The isolated compounds may be considered as prototypes for future anticancer agents capable of selective inhibition of human drug-resistant prostate cancer cells. Note that we could not isolate enough leptogorgins C (**3**) and leptogorgoid A (**4**) to investigate the biological activity of these compounds.

## 3. Materials and Methods

### 3.1. General Procedures

Optical rotation was measured using a PerkinElmer 343 polarimeter. UV spectra were recorded on a Shimadzu UV-1601 PC spectrophotometer. ECD spectra were recorded with an Applied Photophysics Chirascan plus spectropolarimeter. IR spectroscopic data were measured using an IR spectrometer Equinox 55 (Bruker, Ettlingen, Germany) in CHCl_3_. The ^1^H and ^13^C NMR spectra were recorded on a Bruker Avance III-700 spectrometer (Bruker, Ettlingen, Germany) at 700 and 175 MHz, respectively, with Me_4_Si as an internal standard. ESI mass spectra (including HRESIMS) were obtained on a Bruker maXis Impact II Q-TOF mass spectrometer (Bruker Daltonics, Bremen, Germany) by direct infusion in MeOH. Low-pressure column liquid chromatography was performed using silica gel (Sigma-Aldrich Co., St. Louis, MO, USA) and Sephadex LH-20 (Sigma, Chemical Co., St. Louis, MO, USA) columns. HPLC was performed using a Shimadzu Instrument equipped with the differential refractometer RID-10A, a YMC-Pack ODS-A (250 × 10 mm) column (YM Co., Ltd., Kyoto, Japan), and a silica gel column (SUPELCOSIL^TM^, 250 × 10 mm, 5 µm) (Sigma-Aldrich Co., USA). TLC was performed on silica gel plates (5–17 µm, Sorbfil, Russia).

### 3.2. Animal Material

The gorgonian *Leptogorgia* sp. (registration number PIBOC O38-011) was collected by dredging during the 38th scientific cruise of R/V “Academic Oparin”, May 2010, South China sea (09°08′2″ N; 109°01′7″ E, depth 134 m), in Vietnamese waters. A voucher specimen of 038-011 sample is stored in the Marine invertebrate collection of the G.B. Elyakov Pacific Institute of Bioorganic Chemistry FEB RAS (Vladivostok, Russia).

### 3.3. Extraction and Isolation

The EtOH extract of the gorgonian (dry weight 170 g) was concentrated and partitioned between *n*-hexane and aqueous EtOH. The EtOH-soluble material was subjected to column chromatography on a silica gel column using CHCl_3_-EtOH (stepwise gradient, 1:0 1:1). Fractions eluted with CHCl_3_:EtOH (20:1) were concentrated and residue (171.3 mg) was subjected to column chromatography on a LH-20 column using CHCl_3_:EtOH, 2:1 to yield two fractions: F1 (46.6 mg) and F2 (61.4 mg). Preparative HPLC of the fraction F1 (SUPELCOSIL, *n*-hexane:EtOAc, 1:1) gave pure leptogorgin A (**1**, 2.5 mg, 0.002% based on dry weight of gorgonian). Preparative HPLC of the fraction F2 (YMC-Parck ODS-A, EtOH:H_2_O, 3:2) gave three sub-fractions: F2-1 (2.5 mg), F2-2 (6.4 mg), and F2-3 (8.0 mg). Preparative HPLC of the fraction F2-1 (SUPELCOSIL, *n*-hexane:EtOAc, 2:3) gave pure leptogorgin C (**3**, 1.0 mg, 0.001% based on dry weight of gorgonian). Preparative HPLC of the fraction F2-2 (SUPELCOSIL, *n*-hexane:EtOAc, 1:1) gave pure leptogorgin B (**2**, 0.8 mg, 0.001% based on dry weight of gorgonian). Preparative HPLC of the fraction F2-3 (SUPELCOSIL, *n*-hexane:EtOAc, 1:1) gave pure leptogorgoid A (**4**, 0.6 mg, 0.0006% based on dry weight of gorgonian).

### 3.4. Compound Characterization Data

*Leptogorgin A* (**1**): colourless oil; ${\left[\alpha \right]}_{D}^{22}$ +38.7 (*c* 0.2, CHCl_3_); UV (EtOH) λ_max_ (log ε) 195 (4.05), 229 (3.75) nm; ECD (*c* 1 × 10^−3^ M, EtOH) λ_max_ (Δε) 194 (7.56), 228 (9.41), 274 (−3.52), 333 (1.30) nm; IR (CHCl_3_): ν_max_ 3604, 2964, 2928, 2860, 1723, 1641, 1458, 1387, 1365, 1261, 1243, 1104, 1012 cm^−1^; ^1^H and ^13^C NMR data (CDCl_3_), Table 1; HRESIMS *m/z* 273.1459 [M + Na]^+^ (calcd for C_15_H_22_O_3_Na, 273.1461); HRESIMS *m/z* 249.1498 [M − H]^−^ (calcd for C_15_H_21_O_3_ 249.1496).

*Leptogorgin B* (**2**): colourless oil; ${\left[\alpha \right]}_{D}^{22}$ +16 (*c* 0.1, CHCl_3_); UV (EtOH) λ_max_ (log ε) 196 (3.23), 229 (3.07) nm; ECD (*c* 3 × 10^−3^ M, EtOH) λ_max_ (Δε) 197 (2.90), 226 (1.41), 254 (−1.06), 336 (0.43) nm; ^1^H and ^13^C NMR data (CDCl_3_) Table 1; HRESIMS *m/z* 315.1571 [M + Na]^+^ (calcd for C_17_H_24_O_4_Na, 315.1567); HRESIMS *m/z* 291.1602 [M − H]^−^ (calcd for C_17_H_23_O_4_ 291.1602). 

*Leptogorgin C* (**3**): colourless oil; ^1^H and ^13^C NMR data (CDCl_3_) Table 1; HRESIMS *m/z* 273.1463 [M + Na]^+^ (calcd for C_15_H_22_O_3_Na, 273.1461); HRESIMS *m/z* 249.1496 [M − H]^−^ (calcd for C_15_H_21_O_3_ 249.1496). 

*Leptogorgoid A* (**4**): colourless powder; ${\left[\alpha \right]}_{D}^{22}$ +33 (*c* 0.05, CHCl_3_); ^1^H and ^13^C NMR data (CDCl_3_) Table 1. HRESIMS *m/z* 437.3021 [M + Na]^+^ (calcd for C_27_H**_42_**O_3_Na, 437.3026); HRESIMS *m/z* 413.3060 [M − H]^−^ (calcd for C_27_H_41_0_3_ 413.3061).

*MTPA esterification of***1**. To a part of **1** (0.6 mg) in dry C_5_H_5_N (1 µL), *R*-(−)-α-metoxy-α-trifluoromethylphenylacetyl chloride (10 µL) was added. The mixture was stirred on one hour at room temperature.and evaporated in vacuo to give (*S*)-MTPA diester **1a**. By the same procedure, (*R*)-MTPA diester **1b** was prepared.

*(S)-MTPA diester* (**1a**): Select ^1^H NMR data (CDCl_3_) see Table S1. HRESIMS *m/z* 707.25 [M + Na]^+^ (calcd for C_35_H_38_F_6_O_7_Na, 707.25).

*(R)-MTPA diester* (**1b**): Select ^1^H NMR data (CDCl_3_) see Table S1. HRESIMS *m/z* 707.25 [M + Na]^+^ (calcd for C_35_H_38_F_6_O_7_Na, 707.25).

### 3.5. Bioactivity Assay

#### 3.5.1. Reagents

The MTT reagent (Thiazolyl blue tetrazolium bromide) was purchased from Sigma (Taufkirchen, Germany).

#### 3.5.2. Cell Lines and Culture Conditions

The human prostate cancer cells 22Rv1 and human prostate non-cancer cells PNT2 were purchased from ATCC. Cell lines were cultured according to the manufacturer’s instructions in 10% FBS/RPMI media (Invitrogen, Carlsbad, CA, USA) and handled as described in [29].

#### 3.5.3. In Vitro MTT-Based Drug Sensitivity Assay

The in vitro cytotoxic activities of the isolated substances were evaluated by MTT assays. The assays were performed as described previously [30]. In brief, cells were seeded in 96-well plates (6 × 10^3^ cells/well), incubated overnight, and treated with the tested compounds for 72 h. Next, 10 μL/well of MTT reagent was added and the plates were incubated for 2 h. The media were aspirated and the plates were dried. The formed formazan crystals were dissolved in DMSO and the cell viability was measured using an Infinite F200PRO reader (TECAN, Männedorf, Switzerland). Results were calculated by the GraphPad Prism software v. 7.05 (GraphPad Prism software Inc., La Jolla, CA, USA) and are represented as the IC_50_ of the compounds against the control cells treated with the solvent alone.

#### 3.5.4. Colony Formation Assay

Colony formation assay was performed as described before, with slight modifications [30]. Cells were treated with the drug for 48 h; then, cells were trypsinized and the number of alive cells was counted with the trypan blue exclusion assay as described before [31]. One hundred viable cells were plated into each well of 6-well plates in complete drug-free media (3 mL/well) and were incubated for 14 days. Then, the media were aspirated, surviving colonies were fixed with 100% MeOH, followed by washing with PBS and air-drying at RT. Next, cells were incubated with Giemsa staining solution for 25 min at RT, the staining solution was aspirated, and the wells were rinsed with dH_2_O and air-dried. The number of cell colonies was counted with the naked eye.

## 4. Conclusions

In summary, ^1^H NMR-guided chemical investigation led to the isolation of three new humulane-type sesquiterpenoids and one new steroid. The structures of the new compounds were elucidated via analyses of their MS, NMR, and ECD spectroscopic data, as well as using the Mosher’s esters analysis. These molecules represent the new humulenes possessing an oxygenation pattern which was significantly different from those found in plants, liverworts, and fungi. Leptogorgin A (**1**) exhibits a moderate cytotoxicity to human prostate cancer 22Rv1 cells.

## Figures and Tables

**Table 1:** ^1^H (700 MHz) and ^13^C (175 MHz) NMR spectroscopic data for **1**, **2** and **3** in CDCl_3_.

Position	1		2		3	
	δ_C_	δ_H_ mult (*J* in Hz)	δ_C_	δ_H_ mult (*J* in Hz)	δ_C_	δ_H_ mult (*J* in Hz)
1	38.0 C	-	38.1 C	-	40.4 * C	-
2	164.8 CH	6.32, d (16.3)	162.8 CH	6.24, d (16.3)	152.7 CH	6.29, d (16.1)
3	128.1 CH	5.97, d (16.3)	128.1 CH	6.07, d (16.3)	128.4 CH	5.76, d (16.1)
4	200.8 C	-	199.4 C	-	204.3 C	-
5	143.0 C	-	145.2 C		48.6 CH	3.38, m
6	133.8 CH	5.75, d (10.6)	129.5 C	5.70, dt (10.6; 1.3)	41.2 CH_2_	2.43, dd (16.9; 2.9)
						2.73, dd (16.9; 9.7)
7	71.7 CH	4.21 td (10.6; 5.4)	72.9 CH	5.28, td (10.6; 5.1)	204.3 C	
8	45.3 CH_2_	1.96, m	42.7 CH_2_	2.03, m	54.1 CH_2_	3.00, d (12.4)
		2.68, dd (12.2; 5.4)		2.69, dd (12.5; 5.1)		3.15, d (12.4)
9	132.4 C	-	128.1 C	-	127.8 C	-
10	125.9 CH	5.22, brd (12.5)	127.1 C	5.32, m	129.0 CH	5.37, ddd (10.5; 5.7, 1.2)
11	40.7 CH_2_	1.95, m	40.7	1.97, m	40.2 * CH_2_	2.00, m
		2.40, t (12.5)		2.39, t (12.6)		2.07, m
12	64.7 CH_2_	4.25, d (13.3)	64.8 CH_2_	4.26, dd (13.2; 4.6)	63.0 CH_2_	3.78, m
		4.38, d (13.3)		4.40, dd (13.2; 6.3)		3.89, m
13	20.1 CH_3_	1.72, s	20.0 CH_3_	1.73, s	19.0 CH_3_	1.64, s
14	24.0 CH_3_	1.18, s	23.9 CH_3_	1.21, s	28.8 CH_3_	1.21, s
15	29.1 CH_3_	1.13, s	29.2 CH_3_	1.13, s	24.3 CH_3_	1.09, s
COCH_3_			169.7 C	-		
COCH_3_			21.2 CH_3_	1.98, s		

* Signals may be interchangeable.

**Table 2:** ^1^H (700 MHz) and ^13^C (175 MHz) NMR spectroscopic data for **4** in CDCl_3._

Position	δ_C_	δ_H_ mult (*J* in Hz)	Position	δ_C_	δ_H_ mult (*J* in Hz)
1	35.7 CH_2_	1.70, m	16	28.5 CH_2_	1.29, m
		2.03, m			1.70, m
2	34.0 CH_2_	2.34, m	17	55.6 CH	1.19, m
		2.42, m			
3	199.5	-	18	12.2 CH_3_	0.74, s
4	123.8 CH	5.72 s	19	17.4 CH_3_	1.19, s
5	171.4C	-	20	39.8 CH	2.14, m
6	32.9 CH_2_	2.27, ddd (14.7; 4.1; 2.4)	21	20.3 CH_3_	1.04, d (6.6)
		2.40, m			
7	32.0 CH_2_	1.02, m	22	140.8 CH	5.61, dd (8.6; 15.3)
		1.84, m			
8	35.7 CH	1.53, m	23	126.0 CH	5.43, dd (7.3; 15.3)
9	53.8 CH	0.94, m	24	79.7 CH	3.84, d (7.3)
10	38.6 C	-	25	72.8 C	-
11	21.0 CH_2_	1.44, ddd (13.6; 17.1; 4.2)	26	23.8 CH_3_	1.15, s
		1.54, m			
12	39.5 CH_2_	1.20, m	27	26.4 CH_3_	1.20, s
		2.01, m			
13	42.5 C	-			
14	55.8 CH	1.04, m			
15	24.2 CH_2_	1.11, m			
		1.60, m			

## Supplementary Materials

The following are available online at https://www.mdpi.com/1660-3397/18/6/310/s1. Copies of HRESIMS, 1D- and 2D-NMR spectra of **1**–**4**.
